# Comprehensive evaluation of critical-size calvarial defect in athymic rat model

**DOI:** 10.3389/fphys.2025.1662424

**Published:** 2025-09-16

**Authors:** Todd M. Parco, Jon D. Wagner, Pratish Parbhoo, Caroline Soviak, Yuan Hu, John T. Araiza, Colin O'Beirne, Sehrish Javaid, Praveen Parachuru, Matthew Lewis, Carlos A. Acosta, Rajendra Prasad Settem, Toshihisa Kawai, Umadevi Kandalam

**Affiliations:** ^1^ Woody L. Hunt School of Dental Medicine, Texas Tech University Health Sciences Center, El Paso, TX, United States; ^2^ University at Buffalo School of Dental Medicine, Buffalo, NY, United States; ^3^ Nova Southeastern University College of Dental Medicine, Fort Lauderdale, FL, United States; ^4^ L. Frederick Francis Graduate School of Biomedical Sciences, El Paso, TX, United States

**Keywords:** critical size defect, calvarial defect, athymic rat, bone regeneration, musculoskeletal disorders

## Abstract

The critical size defect (CSD), which defines the threshold beyond which spontaneous healing fails, serves as a foundational tool in bone regeneration research. This study investigated calvarial CSDs in an athymic rat model to refine the definition, emphasize its significance, and address inconsistencies in experimental design across existing information. A 4.5 mm diameter calvarial defect was surgically created in athymic rats. Bone regeneration was assessed using micro-computed tomography (micro-CT) at 1, 4, and 8 weeks after the surgery, compared to the baseline (day 0) post-surgery. Histological assessment was performed using a standard hematoxylin and eosin (H&E) staining protocol at 8 weeks post-surgery. Quantitative data from micro-CT analysis revealed no increase in bone volume or percentage volume from day 0 to week 1. We observed bone regeneration initiated at week 4 and progressed through week 8, with healing percentages ranging from 0.1% to a maximum of 7%. Histological examination of tissue at week 8 revealed the presence of loose collagen fibers and interspersed fibroblasts, with no mineralization as observed. These findings confirm that the 4.5 mm defect in calvarial bone qualifies as a critical-size defect in the athymic rat model. The study enhances our understanding of bone healing dynamics in CSDs and provides a validated platform for evaluating novel regenerative therapies.

## 1 Introduction

Bone loss or the absence of bone formation is a persistent clinical issue affecting both humans and other species. Various etiologies of bone loss or impaired formation occur in clinical contexts such as severe trauma, tumor resection, congenital disorders, infections, and systemic disease (Hardy and Cooper, 2009; Roddy et al., 2018; Aalami et al., 2004). When confronted with bone defects, reconstruction of bone is essential to restore skeletal function and integrity. To investigate the clinical applications of bone regenerative therapies, preclinical experimental models have been widely utilized to provide a “proof of principle” (Vajgel et al., 2014). The critical size defect (CSD) model has become an invaluable tool in studying bone regeneration and evaluating the limits of spontaneous healing. A critical size defect is defined as the smallest osseous lesion that will fail to heal spontaneously within the lifespan of the organism, thus necessitating reintervention for successful bone regeneration and healing. Schmitz and Hollinger introduced the CSD concept, emphasizing defect size as a key factor in healing (Schmitz and Hollinger, 1986). The critical size defect model is intended to standardize the testing of bone repair methods and materials that could be used as either alternatives or supplements in existing auto- and allograft materials. By utilizing the CSD model, researchers can provide a broad understanding of the underlying mechanisms of bone formation. Although this model is highly used and has proven to be highly effective in pre-clinical research studies, the concept of the Critical Size Defect model is subject to discrepancies due to variation across studies. Differences in species, age, defect size, and surgical technique affect study outcomes and complicate standardization. Animal models ranging from mice to non-human primates demonstrate differing intrinsic healing capacities. These variations offer valuable insight but hinder efforts to define a consistent “critical” size. Younger animals, in particular, show stronger regenerative abilities than older ones (Vajgel et al., 2014; Cooper et al., 2010) (Table 1).

**TABLE 1:** Comparative summary of critical size defect models in rats.

Reference	Strain	Gender	Age (weeks)	Defect size (mm)	Group size	Time (week)	Pros	Cons
Bateman et al. (2012)	Sprague-Dawley	Male	10	5	10	6	Reliable model for small defects	May spontaneously heal in some cases
Cooper et al. (2010)	Sprague-Dawley	Male	8	2.3	10	6	Good for sub-critical testing	Not a true CSD
Yoon et al. (2007)	NIH-Foxn1rnu	Not specified	16	8	12	12	Immunocompromised model; human adipose-derived stem cell transplantation	High material cost
Subbiah et al. (2021)	NIH-Foxn1rnu	Male	5–7	8	12	8	Immunocompromised model; human bone marrow stromal cells transplantation	Cost and care complexity
Poser et al. (2014)	Wistar	Female	12	5	12	8	Standardized critical side defect model; no spontaneous healing	Cost and technical complexity
Arosarena and Collins (2003)	Fisher 344	Male	Retired Breeder	4	5	8	Defect did not heal spontaneously	Costly, complex, with limited bone gain

A diverse range of animal models, from guinea pigs and mice to rats and even non-human primates, has been employed to investigate the Critical Size Defect (CSD) model (Cooper et al., 2010; Yoon et al., 2007; Spicer et al., 2012; Delgado-Ruiz et al., 2015; Marei et al., 2018; Yu et al., 2008; Voss et al., 2022; Moest et al., 2020) (Table 2). Each species showcases distinct intrinsic healing capabilities, contributing unique insights to the study of bone regeneration. Moreover, the varied sizes of these animal models enable researchers to explore a spectrum of defect sizes, enriching the breadth of experimental investigations. Additionally, the age of the organisms plays a pivotal role in shaping study outcomes. Studies consistently demonstrate that younger organisms exhibit a more robust capacity for healing, often showcasing more complete regenerative responses compared to their older counterparts (Aalami et al., 2004).

**TABLE 2:** Comparative summary of critical size defect models in other animal species.

Animal	Defect size (mm)	Pros	Cons
Mouse (Cooper et al., 2010) (Taguchi and Lopez, 2021) (Harris et al., 2013)	2–5 mm	Low cost, availability of transgenic models, rapid breeding cycle	Very small surgical field, limited defect size
Rabbit (Delgado-Ruiz et al., 2015) (Taguchi and Lopez, 2021) (Lei et al., 2024)	6–15 mm	Suitable for implant testing, easier surgical manipulation than rodents	Higher cost, limited availability of immunocompromised strains
Dog (Marei et al., 2018) (Taguchi and Lopez, 2021) (Huh et al., 2005)	20 mm	Large defect possible, better bone remodeling similarity to humans	Ethical concerns, higher costs, complex care
Goat (Yu et al., 2008) (Taguchi and Lopez, 2021)	8–20 mm	Large cortical defects possible, load-bearing studies	Special housing needs, more labor-intensive
Sheep (Voss et al., 2022) (Taguchi and Lopez, 2021)	20–30 mm	Good translational model for human-sized defects	High maintenance, longer healing time
Pig (Moest et al., 2020) (Taguchi and Lopez, 2021) (Dewey et al., 2021)	10–25 mm	Thicker cortical bone similar to humans	Aggressive behavior, rapid growth may confound results

Another key factor influencing the experimental outcome in critical size defect (CSD) models is the surgical techniques employed. While several techniques have been described in the previous literature for creating the calvarial defect model, with slight variations in execution. However, options may be constrained by the small size, thin bone structure, and anatomical characteristics of the rat calvaria (Cooper et al., 2010). Commonly reported methods include trepanning, drilling, and punching. The trepanning method involves using a trephine bur of a specific diameter to create the defect, offering high consistency and reproducibility—attributes essential for comparative studies (Pereira et al., 2011). The drilling method utilizes a dental or orthopedic drill to achieve the desired defect size, but is highly operator-dependent, often resulting in variability in defect dimensions. The punch method, similar to the trepanning technique, employs a biopsy punch and has also demonstrated consistency and reproducibility (Kasuya et al., 2018).

A consistent priority across all surgical approaches is the preservation of surrounding tissues during the procedure. This is crucial to enable a more accurate assessment of the bone’s intrinsic healing potential, free from extrinsic or iatrogenic interference. Of particular importance is the dura mater—a dense membrane located directly beneath the calvarial bone in rats. As the outermost layer of the meninges, it not only protects the central nervous system but also contributes significantly to the regenerative and developmental processes of the calvaria. Despite its wide usage, inconsistencies persist regarding the exact parameters that constitute a CSD. Variability in species, age, defect size, surgical technique, and healing timelines challenge the establishment of universal standards (Cooper et al., 2010; Spicer et al., 2012; Senos and Hankenson, 2020). For instance, younger animals demonstrate superior regenerative capacity compared to adults, and surgical techniques vary in reproducibility depending on the method used (trephine vs. drill vs. punch) (Hudieb et al., 2021).

In rodent models, particularly rats, critical size calvarial defects are most frequently studied. Traditional practices recognize 5 mm and 8 mm defects as “critical,” yet limited data exists for intermediate or smaller defect sizes (Cooper et al., 2010; Spicer et al., 2012; Senos and Hankenson, 2020). Furthermore, defect size is often reported based on the nominal trephine bur diameter rather than the actual postoperative measurement, contributing further to inter-study variability.

This study aims to clarify and propose standardization for the rat calvarial CSD model by:

• Precisely defining critical defect size based on postoperative measurements.
• Standardizing periosteal removal to eliminate endogenous healing variability.
• Considering age-matching to account for regenerative capacity differences.
• Proposing clinically relevant healing timelines, rather than solely “lifetime” definitions.

Through this comprehensive evaluation, we aim to strengthen the translational relevance of rat calvarial CSD models in bone tissue engineering applications.

## 2 Materials and methods

Buprenorphine (Ethiqa, catalog number 1217793), Lidocaine, 1 wt/vol% with 1:100,000 epinephrine (Henry Schein, catalog number 1047099), Bupivacaine HCL 0.5%. (Covertus, catalog number 054893), Sterile normal saline (Henry Schein, catalog number 6985812), Formalin (Fisher Scientific, catalog number SF100). Isoflurane (Henry Schein, catalog number 1084262), Phosphate buffered saline (Gibco, catalog number 21600010) ethanol, 70% (VWR, catalog number 71001–654), Oxygen, USP Grade (Matheson Tri-Gas), Carbon dioxide, USP Grade (Matheson Tri-Gas).

### 2.1 Equipment

• Electric clippers (Oster, Model A2)
• Lacrilube (Henry Schein, catalog number 3773656)
• Kent Scientific SomnoSuite® Low-Flow Anesthesia System (VetEquip, Tabletop System)
• Rodent heart rate, respiratory rate, temperature, and sp02 monitor (Kent Scientific PhysioSuite)
• Kent Scientific Far Infrared warming pad
• Kent Scientific Preset Pressure Reducer
• Press’n Seal
• Alcohol swabs (Covidien, catalog number 5110)
• Iodine swab sticks (Dynarex, catalog number 1201)
• Sterile, disposable scalpel blade, #15 (Miltex, catalog number 4–115)
• Surgical/dental drill (NSK Surgic XT Plus, catalog number Y141246)
• Contra angle handpiece (NSK Ti-Max, catalog number SG20L)
• Straight handpiece (NSK Ti-Max, catalog number SG65L)
• Dressing Pilers Non-Locking Size DPU17 Serrated Tips (Henry Schein, catalog number 6009863)
• Forceps Size 43 Adson Brown (Henry Schein, catalog number 6003739)
• Adson Tissue Forcep Straight 4–3/4 (Henry Schein, catalog number 9538273)
• Periosteal Elevator Size 14 Goldman-Fox Double End (Henry Schein, catalog number 1003086)
• Carver Size 3-6 Double End Life Steel (Henry Schein, catalog number 5610131)
• Needle Holder Baumgartner German Stainless Steel 5in (Henry Schein, catalog number 1074150)
• Needle Holder Castroviejo Stainless Steel 5.5 in (Henry Schein, catalog number 1070941)
• Scissors 5.5 in Mayo Blunt (Henry Schein, catalog number 1074150)
• Curved Scissor Metzenbaum Blunt (Henry Schein, catalog number 6004211)
• Qulix Periodontal Probe Single End #6 Satin Steel Michigan O (Henry Schein, catalog number 6009943)
• Sponge Bowl Stainless Steel Silver 22oz (Henry Schein, catalog number 4504353)
• Trephine Bur 35 mm 4.6 mm/3.75 mm (Henry Schein, catalog number 1150106)
• Monocryl Suture 5–0 18″Poliglecaprone 25 Monofilament (Henry Schein, catalog number 6541301)
• 18-gauge needle (Becton Dickinson, catalog number 305195),
• 25-gauge needle (Becton Dickinson, catalog number 305122),
• 27-gauge needle (Becton Dickinson, catalog number 305109),
• 26-gauge needle (BD Syringe, Ref 309,625),
• 1 mL syringe (Becton Dickinson, catalog number 309659),
• 5 mL syringe (Becton Dickinson, catalog number 309603)

### 2.2 Animal

**FIGURE 1:**
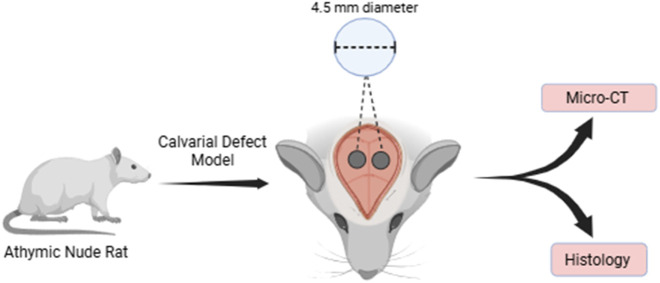
The study design In the rat cranium, two calvarial defects were created, and the bone healing was evaluated using micro-CT—and histological analysis. (Figure made using Biorender software).

All animal procedures were conducted in accordance with the guidelines of the Institutional Animal Care and Use Committee (IACUC Protocol # 22002) of Texas Tech University Health Science Center, El Paso, TX. Eight-week-old rats (300–350 g) purchased from Inotiv (Indianapolis, IN) were brought to the facility and allowed to acclimatize for 1 week in the animal facility, before starting the experiments. Four male Athymic rats (Hsd: RH-Foxn1rnu) were used for this study. The study design was represented in Figure 1.

**FIGURE 2:**
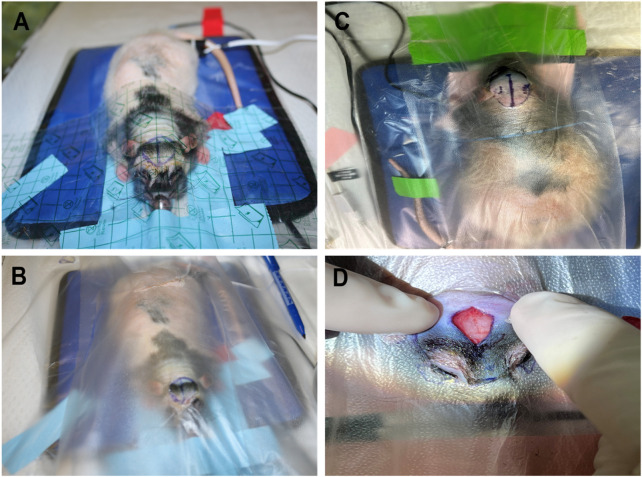
Preoperative preparation: **(A)** The Rat was placed on the surgery table and draped with Tegaderm. **(B)** The Rat’s entire body was covered and prepared for surgery. **(C)** The Incision site was measured and marked. **(D)** The Incision was made.

Preoperative preparation (Figures 2A–D): All surgical instruments were sterilized in an autoclave and instruments were allowed to cool to room temperature (20 °C–25 °C). Operating table was sterilized with cavicide. Rat was placed in an induction chamber and anesthetized using gradually increasing levels of isoflurane (up to 4%) in oxygen until the rat was rendered unconscious. This was determined by the complete loss of its righting ability. Isoflurane was chosen over the commonly used injectable combination of ketamine (80–100 mg/kg and xylazine (0.5–10 mg/kg) for several reasons. The primary advantage of isoflurane is the precise control it offers over anesthetic depth, which is not possible with fixed-dose injectable agents. To ensure adequate pain management, both local anesthetics (lidocaine and bupivacaine) and a systemic opioid were administered. Additionally, the use of inhalant anesthesia ensured that the animal remained fully anesthetized regardless of the procedure’s duration. While this protocol was effective, a multimodal anesthetic approach using both injectable and inhalant anesthetics may be explored in the future to further optimize outcomes.

### 2.3 Surgical draping and stabilization

Each rat was placed in sternal recumbency on a clean far infrared (FIR) warming pad set to maintain the rat’s core body temperature at 37.5 °C. A large sheet of Tegaderm (3M) was then placed partially over the animal’s upper body and over the proposed surgical area. The entire body was not enclosed due to the risk of overheating and to allow for injections or access to the animal during the procedure (Figures 2 A,B). In addition, to maintain sterility and the animal’s position, a sheet of Press’n Seal was applied completely over the animal and the surrounding surgical field.

### 2.4 Procedure

Rats were anesthetized using isoflurane administered via an induction chamber and nose cone (induction at 4%–5%, maintenance at 1.5%–2%). Depth of anesthesia was confirmed by the lack of righting ability, palpebral reflex, and absence of a toe pinch reflex. Immediately upon transfer from the induction chamber to the nose cone, the eyes were lubricated with Lacrilube ointment. Perioperative analgesia was initiated with a subcutaneous injection of long-acting buprenorphine (0.65 mg/kg of body weight) and warm sterile normal saline at 10 mg/kg/h (given as a bolus) to compensate for intraoperative fluid loss. The scalp was shaved from the bridge of the nose to the occipital region using electric clippers, and the area was cleaned with an alcohol swab to remove loose hair. Rats were maintained on a far-infrared heating pad (set at 37.5 °C) throughout the surgery. Core body temperature was monitored using a rectal probe. Oxygen saturation, heart rate, and respiratory rate were monitored via a pulse oximeter placed on the rear foot.

Aseptic preparation of the surgical site included clipping and cleaning of the proposed surgical site. Before the site was scrubbed, a local anesthetic was injected subcutaneously along the dorsal midline along the length of the proposed incision. A 50/50 by volume mixture of 1% lidocaine with 1:100,000 epinephrine and bupivacaine. This combination was used to take advantage of the properties of lidocaine (15–45 min) while also benefiting from the short-acting properties of lidocaine (15–45 min) and the long-acting properties of bupivacaine (4–8 h). The skin was then prepped with alternating passes of povidone-iodine and 70% isopropyl alcohol, three times each, ensuring not to get any into the animal’s eyes. After aseptic preparation, including the aseptic skin preparation and sterile draping with Tegaderm and Press N′ Seal, a midline skin incision approximately 2 cm in length was made along the midline of the calvarium to expose the underlying (Figures 2 C,D). The subcutaneous tissue was bluntly dissected away, and the periosteum was scraped away to expose the coronal sutures and parietal plates.

**FIGURE 3:**
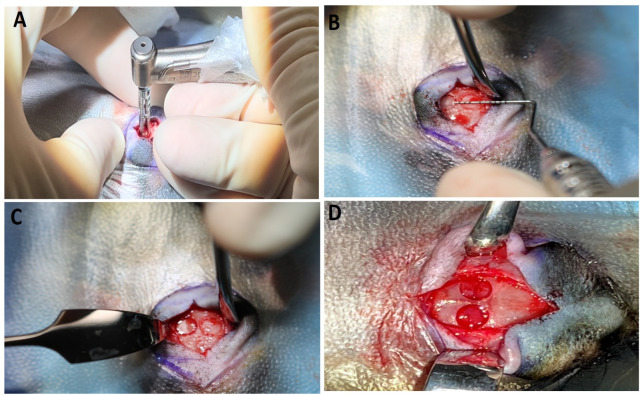
**(A–D)**: Surgical procedure and creating defect: **(A)** Figure showing the process of making defect with 3.75 mm Trephine Bur **(B)** Creation of calvarial defect and measuring with periosteal probe, each defect size was approximately 4.5 mm. **(C)** Two bone defects were made **(D)** The picture showing two bone defects made (magnified view).

To minimize endogenous osteogenic influences, the periosteum was carefully dissected and removed from the calvarium. Two bilateral critical-sized defects (CSDs) were created using a 3.75 mm trephine bur mounted on a contra-angled dental surgical drill, operating at 800 RPM with continuous sterile saline (Figures 3 A–D). Slow speed of trephine and irrigation were maintained as they are critical factors to prevent thermal injury. To preserve the integrity of the underlying dura and avoid potential damage to brain tissue, the trephine bur was used only to thin the calvarial bone without full penetration. Care was taken throughout to prevent injury to the dura mater. Damage to the dura can significantly impair bone regeneration and delay or hinder the healing process within the defect. Once the defect outline was adequately thinned, a surgical blade was inserted circumferentially around the margin to gently separate the bone from the underlying dura, allowing the bone disc to be lifted carefully. The blade was also used to delicately detach any adherent dura mater from the internal surface of the calvarium beneath the defect. Gentle downward pressure was applied while drilling to create each defect. Margins were examined for residual bone fragments, and defects were thoroughly irrigated with sterile saline. Each defect was standardized to a diameter of 4.5 mm, measured using a periosteal probe, and the measurement was verified using precision calipers postoperatively. A second defect was created on the same calvarium, separated from the first by an appropriate bony bridge to maintain mechanical integrity. The midline was avoided to avoid penetration of the sagittal sinus, a large venous channel of the (Figures 3 C,D). The total surgery time to create one defect took approximately 30 min.

### 2.5 Wound closure and recovery

Following defect creation, closure of the periosteum and subcutaneous tissue was performed with 5–0 monocryl sutures in a simple interrupted or simple continuous pattern. The skin was repositioned and closed with either a simple continuous or a simple interrupted pattern using 5–0 monocryl suture material.

### 2.6 Postoperative care

After surgery, the rats’ heads were cleaned with saline to remove any blood from the surgical area. Once the rats began showing signs of purposeful movement, they were transferred to a warmed incubator with supplemental oxygen. Animals were returned to their cages with *ad libitum* access to food, water, and Recovery Diet Gel cups. Animals were housed separately for at least 24 h to allow for undisturbed recovery. Afterward, the rats were transferred to regular husbandry cages with their cage mate. The health status of the animals was observed twice a day for 7 days after surgery, and then once daily for 2 weeks. During this period, their appearance, weight, activity, behaviors (including indications of pain or discomfort), and food and water intake were observed and recorded until the scheduled euthanasia procedure.

### 2.7 Euthanasia of athymic rats and tissue harvest

At 8 weeks postoperatively, athymic rats were euthanized to facilitate tissue harvest and further analysis. The procedures were conducted in compliance with institutional IACUC guidelines and NIH guide for the Care and Use of Laboratory Animals for humane animal sacrifice and included a secondary method to ensure death.

### 2.8 Anesthesia and primary euthanasia method

Each rat was placed into an isoflurane induction chamber and anesthetized with 4% isoflurane in oxygen until complete anesthesia was achieved, verified by the absence of pedal and corneal reflexes. Once anesthetized, the flow of oxygen was ceased, and carbon dioxide was introduced at a flow rate of 8–12 L/min for a minimum of 5 min or until no respiratory movement was observed for at least 1 min, indicating cessation of spontaneous respiration.

### 2.9 Secondary euthanasia method

Following CO2 asphyxiation, Bilateral Thoracotomy was employed to ensure death in accordance with NIH recommendations of a secondary physical method. The animal was removed from the chamber, and bilateral thoracotomy was performed by piercing the intercostal spaces on both the left and right sides of the thorax using a scalpel to collapse the lungs.

### 2.10 Microcomputed tomography analysis micro-CT)

Qualitative and quantitative bone morphometry analyses were performed on both live and euthanized rats using a high-resolution micro-computed tomography (micro-CT, SkyScan 1,276; Bruker, Belgium). The animals were scanned with a rotation step of 0.400°, step and shoot, 360° scanning. Resolution was set to 1024 x 1024, using an aluminum-copper filter. Bone healing was monitored at 0-, 1-, 4-, and 8-week post-surgery using micro-CT. The cross-sectional views of digitally captured images were processed by the NRecon program (Bruker). Three-dimensional images were generated and analyzed by Bruker’s CTVox and CTAn software, respectively. The defects with 4.5 mm diameter were determined according to the region of interest. Bone volume was measured (mm^3^) and expressed as a percentage of total tissue volume. Additionally, trabecular thickness (Tb Th), Trabecular separation (Tb Sp), and trabecular number (Tb N) were measured and compared across day 0, day 1-, 4-, and 8-week time points.

### 2.11 Histology

The calvarial region involving the bone defect site and adjoining tissue was excised from a euthanized rat at 8 weeks and fixed in 10% phosphate-buffered formalin. The collected samples were decalcified and sectioned in paraffin blocks for Hematoxylin and Eosine (H&E) staining (UT Southwestern University histology services, Dallas, TX). The digital images were examined, and histo-morphometric analysis was conducted.

Statistical analysis: The data were expressed in mean standard deviation. Statistical analysis was performed using a one-way analysis of variance (ANOVA) with a multiple comparison Tukey test in GraphPad Prism 10 (GraphPad Software, Inc., La Jolla, CA). The criterion for statistical significance was p < 0.05.

## 3 Results

Animals: All animals survived until 8 weeks. There were no injuries to the dura while creating the defect. The wound area showed no signs of inflammation or adverse reactions after surgery.

**FIGURE 4:**
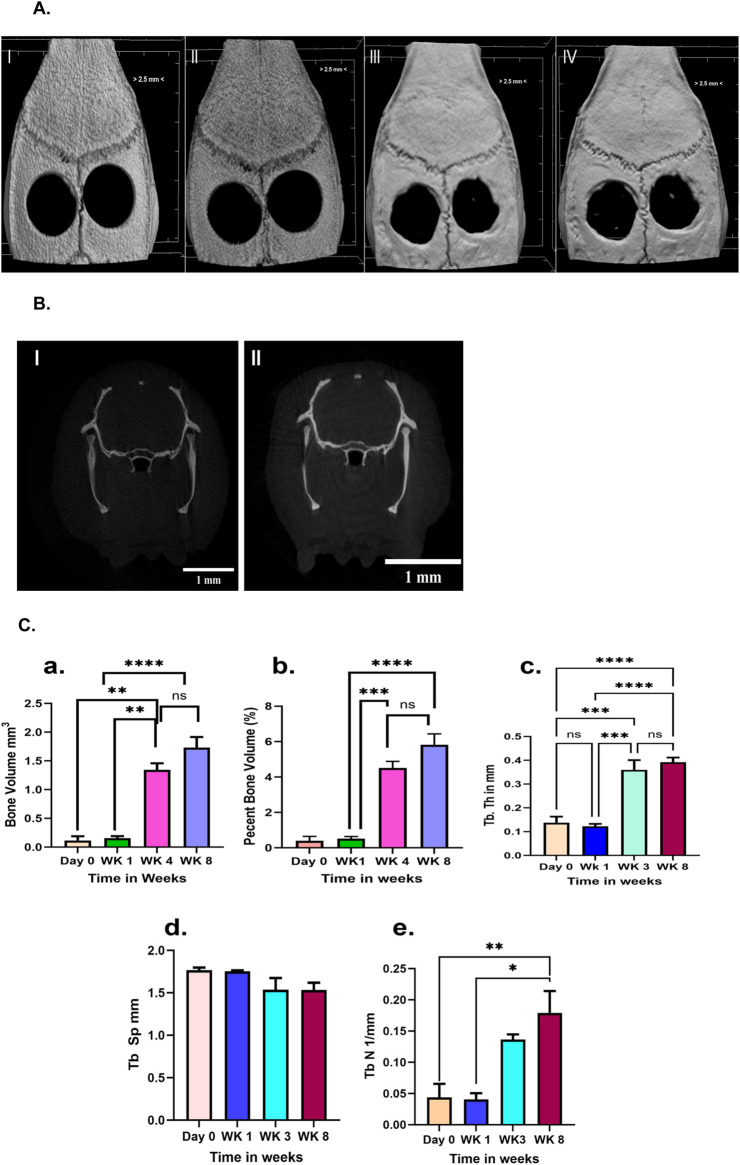
**(A)** Microcomputed images (micro-CT) data showing defect group. A surgical defect was created in the calvarial region. l) Day 0 ll) Week 1 lll) Week 4 lV) 8 weeks after surgery. Scale bar <2.5 mm. **(B)** Trans-axial view: a defect is seen on both sides. l) Day 0, ll) Week 8. Figures showing that there was no spontaneous bone regeneration in the defect area at either 4 or 8 weeks. **(C)** Figure showing quantitative data from micro-CT. **(a)** The bone volume **(b)** BV/TV, **(c)** Tb Th, **(d)** Tb Sp, and **(e)** Tb N at day 0, week 1,4, and 8 weeks after the surgery were measured. Tb Th, trabecular thickness; Tb Sp, trabecular separation; and Tb N, trabecular number. Bone volume and percent volume showed a significant increase (P < 0.05) from week 1 to week 4 post-surgery. However, there was no significant improvement in healing between weeks 4 and 8. Tb Th showed a trend towards increasing from day 0 to week 8, while Tb Sp decreased accordingly. On the other hand, Tb N increased significantly from day 0 to week 8. Overall data showed a significant increase in bone healing from day 0 to week 4 and week 8, as well as from week 1 to week 4 and week 8. The data indicated that no spontaneous healing occurred until 8 weeks post-surgery. A one-way ANOVA with a Tukey multiple comparison test was conducted for statistical analysis. **P < 0.05, ns not significant.

Micro CT: Micro-CT was employed to obtain 3D reconstruction images for characterizing the quality and quantity of bone healing and newly formed bone (Bouxsein et al., 2010). The qualitative evaluation of spontaneous bone healing ability and comparison among the groups demonstrated that bone healing began at the edges of the defect (Figures 4A–C). However, over the course of 8 weeks, the defect was not completely healed, indicating that it was of a critical size. Figure 4B shows the trans-axial view (2-D) of bone healing at Day 0 and Week 8, showing that no considerable bone healing occurred until Week 8. Day 0 to Week. Figure 4C shows the quantitative data for bone volume (mm^3^), percent bone volume, trabecular thickness (Tb Th), Trabecular space (Tb Sp), and trabecular number (Tb N). The quantitative data on bone volume and percent volume showed that there was no significant increase from day 0 to week 1. Bone healing began at week 4 and continued until week 8. Most of the healing occurred around the perimeter, and the central area remained empty. The percentage of bone healing ranged from 0.1 to a maximum of 7% among all the rats, except for one defect, where the maximum healing occurred at 11.49% (data not shown). Trabecular thickness and trabecular number significantly increased (P < 0.05) compared to day 0 or day 7 to 4 weeks and 8 weeks, while trabecular space showed a slight decrease. However, there was no significant change throughout the time. The trabecular thickness increased by over 2.5-fold at 4- and 8-week post-surgery compared to day 0, while the trabecular number showed approximately a fourfold increase in the 4-week and 8-week groups compared to day 0.

**FIGURE 5:**
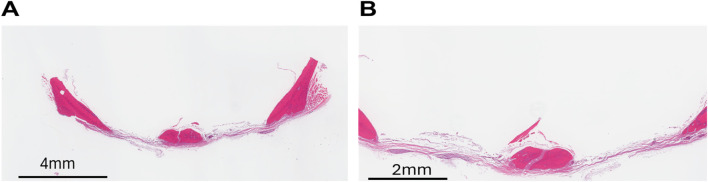
Histological analysis demonstrated that there was no spontaneous bone healing in the defect group. This confirms that we created a critical size defect. **(A)** ×2 magnification showing both sides of the defect, 8 weeks post-surgery. **(B)** ×4 magnification.

Histology: Histological data provide two-dimensional measurements (Dempster et al., 2013). We collected data at the end of the study period. The rats were euthanized at the end of the study period (8 weeks). The histological sections were stained with H&E. The defect showed deposition of connective tissue consisting of fibroblasts and loose collagen fibers. Areas of necrotic bone without osteocytes were seen in some samples. Except in two defects where there was little bone formation observed, no areas of newly forming bone were identified (Figures 5A,B). The areas where small bone formation occurred were towards the periphery, which is in agreement with our micro-CT results. Overall results showed that there was no functional bone formation at the end of 8 8-week period, indicating that the defect made was of critical size.

## 4 Discussion

This study presents a rat model for critical-size calvarial defects, standardized to improve reproducibility and translational potential in bone tissue engineering research. The goal of this study was to provide a critical size defect model that will be useful for bone regenerative therapies. To ensure compatibility with future phases of this research involving xenogeneic cell transplantation, we selected athymic rats as our animal model. Athymic (nude) rats lack a thymus and are therefore immunodeficient, making them incapable of mounting a T-cell–mediated immune response. This unique immunological profile allows for the engraftment and survival of human-derived stem cells. This continuity in model selection improves the translational relevance of our findings and facilitates comparison between scaffold-only and stem cell-augmented healing outcomes (Macchiarini et al., 2008; Bugelski and Martin, 2012).

Although we followed standard surgical techniques, keeping animal welfare in mind, we made some improvisations on specific surgical techniques. A 2 cm midline incision was made along the calvarium, undermining to expose coronal sutures and parietal plates. The incision length was selected to allow sufficient tissue retraction without interfering with the cutting edge of the trephine burr. We selected not to use the L-shaped incision as previously reported due to its association with increased inflammation and a higher risk of tissue damage associated with longer incisions.

Another key modification in our surgical technique was to create a standardized surgical field and minimize endogenous sources of osteogenesis. To achieve this, the periosteum was carefully dissected away from the calvarium prior to defect creation. The periosteum is a highly vascularized membrane rich in mesenchymal stem cells, known to contribute significantly to calvarial bone regeneration through both direct osteoprogenitor activity and the secretion of pro-angiogenic and osteoinductive factors, such as vascular endothelial growth factor (VEGF) and bone morphogenetic proteins (BMPs) (Colnot, 2009; Bouletreau et al., 2002). Previous studies have shown that spontaneous healing in critical-sized defect models can be influenced by periosteal preservation, thereby introducing variability that may confound the interpretation of experimental interventions (Histing et al., 2011; Schindeler et al., 2008). By removing the periosteum, we aimed to eliminate this source of variability and improve internal consistency across experimental groups.

The rodent critical size defect (CSD) model is well-established in bone regeneration research, with adequate data characterizing the healing pattern in defects that exceed defined thresholds. In particular, standardized models frequently define CSDs as 8 mm defects in rat calvaria and 5 mm in mouse calvaria, as these sizes consistently fail to heal spontaneously within the animal’s lifetime (Schmitz and Hollinger, 1986). While numerous studies have explored these defect sizes, there is a notable lack of evidence and consensus regarding the healing response in sub-critical defects—those smaller than the established CSD thresholds. Although many publications reference the concept of a “critical size,” the literature lacks a universally accepted percentage-based threshold for defining criticality. This is primarily due to variations in experimental design, animal models, anatomical sites, and healing assessment methodologies across studies, which complicate direct comparisons and the establishment of standardized criteria. The goal of this article is to provide a standardization of the CSD model in the context of defects, while emphasizing clinical relevance. Thus, we have chosen to investigate the time course of spontaneous bone healing and evaluate whether the defect size that we made was critical. In our study, we considered day 0 as our baseline (Control) and evaluated bone healing.

While many studies define defect size based on the bur used, we measured each defect postoperatively with precision calipers. This revealed that a head diameter of 3.75 mm (inner diameter) bur consistently produced 4.5 mm (with 4.75 mm outer diameter) diameter defects, likely due to bur geometry, drilling pressure, and microfracture at the margins. Vajgel et al. highlighted these inconsistencies, noting that while a 5 mm defect is often considered appropriate for the CSD model in rodents, further studies are necessary to establish standardized criteria (Vajgel et al., 2014). Accurate measurement is crucial in determining whether a defect meets the criteria for critical size. Table 3 represents the studies which has mentioned the bur size.

**TABLE 3:** Showing available literature on the studies that reported the actual bur size.

Defect size (mm)	Authors	Year	Article title
5.0	Zou et al. (Zou et al., 2011)	2011	Repair of Critical-Size Rat Calvarial Defects Using Genetically Engineered Bone Marrow-Derived Mesenchymal Stem Cells Overexpressing Hypoxia-Inducible Factor-1α
5.0	Stephan et al. (Stephan et al., 2010)	2010	Injectable Tissue-Engineered Bone Repair of a Rat Calvarial Defect
5.0	Honda et al. (Honda et al., 2013)	2013	Bone tissue engineering with bone marrow-derived stromal cells integrated with concentrated growth factor in *Rattus norvegicus* calvaria defect model
8.0	Spicer et al. (Spicer et al., 2012)	2012	Evaluation of Bone Regeneration Using the Rat Critical Size Calvarial Defect

In contrast to studies that report defect size based on trephine bur diameter, we utilized a 3.75 mm round bur to create defects that were consistently measured postoperatively as 4.5 mm in diameter using precision calipers. We confirmed the diameter of the defect utilizing the ROI in micro-CT. The discrepancy between bur diameter and final defect size is attributable to several factors, including bur geometry, drilling angle, pressure during surgery, and minor bone fragmentation at the edges of the defect (Hidalgo and Pusic., 2002; Schmitz and Hollinger, 1986). For this reason, all measurements reported in this study reflect the actual post-surgical defect dimensions rather than the nominal bur size. This distinction is crucial in determining what qualifies as a critical-sized defect and in comparing results across studies. Due to the geometry of trephine burs, an 8 mm trephine bur will make a defect larger than 8 mm. Previous studies reported the size of the bur and not the post-operative defect size (Spicer et al., 2012). Bur size doesn’t accurately represent the true size of the defects, which could create significant discrepancies in the study results. Such inconsistencies have been underreported in the literature, despite their potential impact on the reproducibility and interpretation of bone healing outcomes in cranial defect models.

Micro-CT analysis revealed that bone formation was not uniform throughout the defect, more towards the periphery not in the center. Our results revealed that there was no spontaneous healing until 8 weeks, indicating that the defect we made was a critical-size defect. The percent bone formation has not exceeded beyond—7%, except in one defect (11.49%). A 2023 study by Qabbani et al. found an average healing rate of 10% at the control defect site with no intervention (Al Qabbani et al., 2023). This study used 24 Sprague-Dawley rats with a similar 8-week period, however, a 5 mm defect.

In contrast with the micro-CT data, Histological analysis at 8 weeks post-surgery revealed no evidence of bone formation. Histologically, we observed the presence of dense fibrous tissue interspersed with fibroblastic cells, with a little or no mineralization. This discrepancy can be attributed to the presence of small and scattered mineral deposits that are sufficient to generate a micro-CT signal but are lost during the decalcification process used for histological examination. Study limitations also include a relatively small sample size and the restriction of histological assessment to a single time point—8 weeks post-surgery.

The recent advances in the scientific field, offering NAMs (new approach methods) such as organoids and organ-on-a-chip technologies, hold significant promise as alternatives to traditional animal surgical methods (Zhou et al., 2025a). Artificial intelligence (AI)-based technology, a game-changer in drug discovery, has ushered in human-relevant research that has entered clinical trials (Zhou et al., 2025b; Liu et al., 2025). Organoids and Organ-on-Chip (OoCs) each offer unique advantages, such as cellular fidelity and ease of implantation (Liu et al., 2025). While organoids provide several benefits, such as cellular fidelity and ease of implantation, they differ from natural organs in that they lack vascularization and standardization. Especially in bone regenerative therapies, vascularization plays a crucial role. Furthermore, animal model-based testing of monoclonal antibodies and biologics has been particularly challenging as species-specific targets and immunogenicity limit the predictive power of animal models. In this scenario, animal models remain essential to providing a systemic environment of a whole organism.

Thus, our overall results support the assertion that 4.5 mm calvarial defects in athymic rats fail to heal spontaneously over 8 weeks. Minimal bone fill and lack of bridging on histology confirm this. These findings align with and build upon earlier reports, underscoring the importance of both measurement standardization and periosteum removal in CSD models.

## 5 Conclusion

Thus, we developed the critical size calvarial defect model. This study demonstrates the successful creation of critical-size calvarial defects in the athymic rat model, which will be favorable for testing the vascularized cell-scaffold system. These results support the validity of our model for evaluating bone regeneration strategies and biomaterial performance in future preclinical studies.

## Data Availability

The raw data supporting the conclusions of this article will be made available by the authors, without undue reservation.
